# High OCT4 Expression Might Be Associated with an Aggressive Phenotype in Rectal Cancer

**DOI:** 10.3390/cancers15143740

**Published:** 2023-07-23

**Authors:** Lina Lambis-Anaya, Mashiel Fernández-Ruiz, Yamil Liscano, Amileth Suarez-Causado

**Affiliations:** 1 Grupo Prometeus & Biomedicina Aplicada a las Ciencias Clínicas, Facultad de Medicina, Universidad de Cartagena, Cartagena 130014, Colombia; llambisa1@unicartagena.edu.co (L.L.-A.); mfernandezr1@unicartagena.edu.co (M.F.-R.); 2 Grupo de Investigación en Salud Integral (GISI), Departamento Facultad de Salud, Universidad Santiago de Cali, Cali 760035, Colombia; yamil.liscano00@usc.edu.co

**Keywords:** rectal cancer, colorectal cancer, cancer stem cells, OCT4, oncofetal protein

## Abstract

**Simple Summary:**

The oncofetal protein OCT4 is a factor that promotes self-renewal and maintenance of pluripotency of embryonic stem cells and induced stem cells, which has been linked to neoplastic processes, but its role and clinical significance in rectal cancer are unknown. Therefore, the aim of this study was to evaluate the expression of the stem cell marker OCT4 related to clinical-pathological characteristics and its clinical significance in rectal cancer patients. Protein expression of the stem cell marker OCT4 was found in rectal tumor tissue but not in adjacent non-tumor tissue, and high expression was significantly associated with phenotypical characteristics of more aggressive rectal cancer.

**Abstract:**

Rectal cancer (RC) is one of the most common malignant neoplasms, and cancer stem cells (CSCs) of the intestinal tract have been implicated in its origin. The oncofetal protein OCT4 has been linked to neoplastic processes, but its role and clinical significance in RC are unknown. This study investigates the expression of the stem cell marker OCT4 related to clinical-pathological characteristics and its clinical significance in RC patients. The expression level of stem cell marker OCT4 was analyzed in 22 primary rectal tumors by western blot. The association between OCT4 protein expression and the clinical-pathological features of tumors was evaluated by χ^2^ test and Fisher’s exact test. We demonstrated that the expression of the stem cell marker OCT4 was observed in tumor tissue but not adjacent non-tumor tissue. High expression of the stem cell marker OCT4 was significantly associated with histological differentiation grade (*p* = 0.039), tumor invasion level (*p* = 0.004), lymph node involvement (*p* = 0.044), tumor-node-metastasis (TNM) stage (*p* = 0.002), and clinical stage (*p* = 0.021). These findings suggest that high OCT4 expression is associated with a more aggressive RC phenotype, with a greater likelihood of progression and metastasis. These results shed light on the importance of targeting this CSC marker to attenuate RC progression.

## 1. Introduction

Colorectal cancer (CRC) is the second leading cause of cancer-related deaths and the third most common cancer worldwide. Rectal cancer (RC) represents 63% of all CRC cases and approximately 58% of deaths caused by this disease globally [1]. Although often considered the same pathological entity, colon and rectal cancers have anatomical and biological differences that affect the prognosis [2,3]. Despite advances in surgical treatment, radiation therapy, chemotherapy, and adjuvant therapies, the prognosis for RC patients is unsatisfactory due to the high rate of local recurrence, treatment resistance, and distant metastasis, which are strongly related to mortality [4].

Cancer stem cells (CSCs) represent a subpopulation of tumor cells characterized by their ability to self-renew, heterogeneity, plasticity, and infinite proliferation. These characteristics, together with scientific evidence, have closely linked this cell population to tumor development, therapy resistance, metastasis, and recurrence after primary treatment [5,6].

Among the transcription factors described as regulators of pluripotency and maintenance of CSCs is the octamer-binding protein 4 (OCT4; also known as POU domain, class 5, transcription factor 1 (POU5F1)), an oncofetal protein that promotes self-renewal and maintenance of pluripotency of embryonic stem cells and induced stem cells, and which has been found to be involved in the progression and poor prognosis of various cancers [7], including gastric [8,9,10], pancreatic [11], breast [12], bladder [13], ovarian [14], prostate [15], and hepatocellular carcinoma [16]. However, few studies have included OCT4 evaluation in RC [17,18], and even fewer have evaluated its clinical significance in these patients [19]. The few existing reports on OCT4 expression have been developed in in vitro models of CRC [20] or in intestinal tumor tissues that have evaluated colon and rectal cancer as the same pathological entity [21,22]. Therefore, the purpose of this study was to investigate the expression of the stem cell marker OCT4 related to clinical-pathological features and its clinical significance in RC patients.

## 2. Materials and Methods

### 2.1. Participants and Sample Collection

A cross-sectional study was conducted that included samples of tumor tissue and adjacent non-tumor tissue obtained from 63 patients diagnosed with primary RC who were treated at two tertiary referral centers in the city of Cartagena, Colombia. A total of 22 samples that met the appropriate size criteria were used for molecular analysis. None of the patients had received neoadjuvant therapy or had a history of other tumors or serious infections. The collected fresh tissues were embedded in RNAlater™ and stored at −80 °C for subsequent analysis. This study was approved by the Ethics Committee of the Universidad de Cartagena (Minutes No. 108, 10 May 2018) and was conducted in accordance with the principles of the Helsinki Declaration. Each eligible participant signed an informed consent form.

### 2.2. Data Collection

Sociodemographic, pathological, and clinical characteristics were collected from medical records and a structured survey. Data such as age, sex, symptoms and clinical history, tumor differentiation grade, lymph node involvement, presence of metastasis, and TNM stage were collected. TNM stage was evaluated according to the seventh edition of the cancer staging manual of the American Joint Committee on Cancer (AJCC) [23].

### 2.3. Western Blot Analysis

The OCT4 protein was isolated using the western blot technique. The tissues were thawed and resuspended in lysis buffer [20 mM Tris-HCl (pH 7.4), 150 mM NaCl, 10% glycerol, 0.2% Nonidet P-40, 1 mM EDTA, 1 mM EGTA, 1 mM phenylmethylsulfonyl fluoride (Sigma-Aldrich, St. Louis, MO, USA), 10 mM NaF, 5 mg/mL aprotinin (Sigma-Aldrich), 20 mM leupeptin (Sigma-Aldrich, St. Louis, MO, USA), and 1 mM sodium orthovanadate (Sigma-Aldrich, St. Louis, MO, USA)]. The concentration of total protein in the supernatant was quantified using a spectrophotometric method [24]. The absorbance at 595 nm was calculated using a standard curve previously prepared with bovine serum albumin (BSA). The samples were prepared with Laemmli loading buffer and denatured by heating at 95 °C for 5 min. A total of 30 μg of protein was loaded onto a 10% polyacrylamide gel prepared with sodium dodecyl sulfate (SDS-PAGE) and subjected to electrophoresis in the presence of an electrophoresis buffer at a constant voltage [25]. After electrophoresis, the proteins were transferred from the gel to a PVDF membrane (iBlot™ Transfer Stack, PVDF Invitrogen™ Thermo Waltham, MA, USA) using the iBlot™ 2 Gel Transfer Device dry transfer technology (Thermo Scientific™, Waltham, MA, USA). Ponceau staining was performed to confirm that the transfer was successful. Subsequently, the membrane was treated with a blocking solution of 5% skim milk in TTBS 1X [10 mM Tris/HCl, 150 mM NaCl, 0.05% Tween-20 (pH 7.5)] and then incubated overnight with the primary antibody anti-OCT4 diluted 1:2000 (Abcam, Cambridge, UK) [EPR2054] (ab109183). After the incubation period, the membrane was washed with TTBS 1X to remove excess antibody. The membrane was then incubated with a horseradish peroxidase-conjugated secondary antibody for 2 h. Finally, the membrane was washed with TTBS 1X and immunodetection was performed using the SuperSignal™ West Pico chemiluminescent substrate (Thermo Scientific™, Waltham, MA, USA). The results were validated using anti-beta glucuronidase (GUSB) antibody 1:2000 (Abcam Cambridge, UK) [EPR10616] (ab166904) as a housekeeping antibody. The PVDF membranes were analyzed using an imaging documentation system, using the iBright CL1000 equipment (Thermo Scientific™ Waltham, MA, USA). The iBright analysis software desktop version (Thermo Scientific™ Waltham, MA, USA) was used to measure the band densitometry to determine OCT4 expression.

### 2.4. Statistical Analysis

Statistical data were analyzed using SPSS for Windows, version 21.0 (IBM Corp., Armonk, NY, USA), and GraphPad Prism 8.0.2 (Graphpad Software Inc., San Diego, CA, USA). The normality of the data distribution was evaluated using the Kolmogorov-Smirnov test. Descriptive data are presented as mean ± standard deviation (SD) or frequency and percentage. Student’s *t*-test was used to determine statistical significance when comparing two groups. To evaluate the association between OCT4 expression and clinical and pathological characteristics, Pearson’s chi-square test or Fisher’s exact test was used. A *p* < 0.05 was considered statistically significant.

## 3. Results

### 3.1. Characteristics of the Studied Population

The average age of the participants was 61.7 years (range between 22 and 90 years), 60.3% (*n* = 38) were women, and 39.7% (*n* = 25) were men, mainly residing in urban areas (74.6%, *n* = 47). The most frequent clinical characteristics were rectal bleeding (52.4%; *n* = 33) and exophytic lesions detected by colonoscopy (57.1%; *n* = 36). The most frequent histological type, histological grade, clinical stage, and TNM were adenocarcinoma (87.5%; *n* = 55), moderately differentiated grade (44.4%; *n* = 28), advanced/regional stage (65.1%; *n* = 41), and classification IIIB (22.2%; *n* = 14) and IVA (17.5%; *n* = 11), respectively (Table 1).

### 3.2. Molecular Determination of OCT4 in Tumor Tissue and Adjacent Non-Tumor Tissue

**Figure 1:**
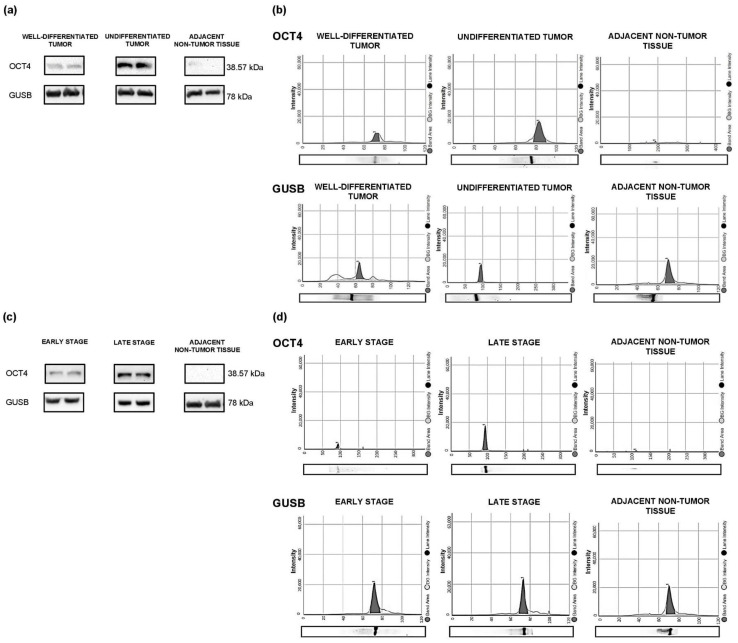
Expression of OCT4 protein in RC tissue and adjacent non-tumor tissue according to the histological differentiation grade and tumor stage. (**a**) Western blot analysis of OCT4 protein expression according to the histological differentiation grade. (**b**) Immunoelectrophoretic analysis of OCT4 protein expression according to the histological differentiation grade. (**c**) Western blot analysis of OCT4 protein expression according to the tumor stage. (**d**) Immunoelectrophoretic analysis of OCT4 protein expression according to the tumor stage. The expression of GUSB was used as a normalizer. See Supplementary Material for the original image of the Western Blots.

The expression of OCT4 protein was higher in tumors with some degree of undifferentiation (Figure 1a,b) and in advanced clinical stages (Figure 1c,d). No protein expression of OCT4 was observed in adjacent non-tumor tissue.

**Figure 2:**
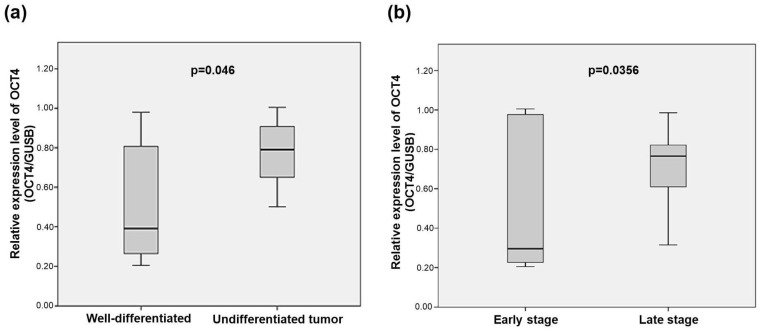
Protein expression levels of OCT4 in rectal tumors. (**a**) OCT4 expression levels in rectal tumors according to histological differentiation grade. (**b**) OCT4 expression levels in rectal tumors according to clinical-pathological stage. Results are the ratio of OCT4 expression normalized to GUSB protein levels. Statistical differences between groups were evaluated by two-tailed Student’s *t*-test.

Bivariate analysis showed higher levels of OCT4 expression in tumors with some degree of undifferentiation (moderately and poorly differentiated) compared to well-differentiated tumors (*p* = 0.046) (Figure 2a). In advanced clinical stages, there was also higher OCT4 expression compared to early stages (*p* = 0.0356) (Figure 2b).

### 3.3. Association between OCT4 Expression and Clinical and Pathological Characteristics

The results showed a significant association between the expression (high/low) of OCT4 and the degree of histological differentiation (*p* = 0.039), invasion level (*p* = 0.004), and lymph node involvement (*p* = 0.044). No association was found between OCT4 expression and tumor metastasis. Significant differences were found in relation to TNM stage (*p* = 0.002) and early clinical stage compared to advanced stage (*p* = 0.021) (Table 2).

## 4. Discussion

Association between stem cell molecules and their derived signals with the evolution of the tumorigenic process is widely accepted [26,27]. It has been proposed that intestinal stem cells represent an important part of the origin of CRC [28,29]. Embryonic stem cells express diverse proteins, including the octamer-binding protein 4 (OCT4), an oncofetal protein that plays a significant role in self-renewal and pluripotency [30]. Recent evidence shows increasing numbers of early-onset RC [31], as well as a high frequency of RC among individuals diagnosed with CRC in the Colombian Caribbean region, as we have previously reported [32]. Therefore, it is pertinent to have more knowledge of the development of this disease to provide new therapeutic targets that allow better management of patients. This study is the first in Colombia to evaluate the relationship between OCT4 expression and clinical-pathological characteristics in primary rectal tumors.

The samples collected for this study were from individuals with an average age of 61.75 years, similar to the mean age reported in epidemiological studies that have evaluated colon and rectal cancer together [33]. Recent studies show increasing incidence rates in adults under 50 years old (early-onset tumors) [34], and in RC, the behavior is similar, including the population of Latin America and the Caribbean [31,35,36]. Our study reports 27% of individuals with RC under 50 years old, which could be supported by the adoption of dietary patterns characterized by high consumption of processed and refined foods and a sedentary lifestyle, among other unhealthy habits that are considered risk factors for the appearance of RC. We found that the incidence was higher in females, consistent with the research of Vargas Moranth et al. in a population of the Colombian Caribbean [37]. Similarly, most individuals were of black race, a characteristic that, beyond being related to a genetic component, is likely to be a consequence of the predominance of this race in the region where the study was carried out, where there was historically African settlement during the colonial period. All these conditions associated with lifestyle and environmental or hormonal factors can impact the clinical characteristics of the population and influence the behavior of the disease. However, it must be borne in mind that all these data related to the epidemiological characteristics of the population should be interpreted with caution, considering that the sample size limits the statistical efficiency of the results.

Our results showed an absence of modulation of the expression of the stem cell marker OCT4 in adjacent non-tumor tissue, but did show OCT4 expression in tumor tissue consistent with other authors, who have reported OCT4 protein expression in various human cancer tissues such as stomach [9], pancreas [11], bladder [13], ovary [14], prostate [15], and CRC, but not in normal somatic tissues [38]. It is believed, then, that OCT4 reactivation occurs in cells that have undergone malignancy [30], in which the expression of pluripotency genes by stem cells has been related to tumor proliferation, metastasis, and poor prognosis [10]. In this sense, our findings suggest that the OCT4 protein may have a relevant role in the development and progression of the tumor.

Likewise, our work shows increased expression of the intestinal oncofetal protein OCT4 in tumors with some degree of undifferentiation (moderately and poorly differentiated), which could lead to progression and even metastasis in RC, considering that it has been reported that the level of stem cell protein expression may be related to the content of these cells in the tumor and suggests its aggressiveness according to the degree of histological differentiation [39]. Similar observations to our data have been described in RC [19], in a case report of CRC [40], as well as in cervical tumors [41] and gastric cancer [8], which could indicate the role of this cell subpopulation in the loss of colonic identity and poorer prognosis [42].

For its part, the data from this study revealed a close association between the expression of the oncofetal protein OCT4 and the stage of the tumors. In this regard, Shaheen et al. stated that higher expression of OCT4 is associated with more advanced stages of CRC and distant metastasis [38]. In line with this, Roudi et al. evaluated, through immunohistochemical staining, the expression of CSCs markers OCT4 and NANOG, reporting a trend between low OCT4 expression and absence of metastasis or lymph node involvement, which could indicate that increased OCT4 expression would contribute to the malignant behavior of CRC and be related to advanced disease [21]. Similarly, several studies have confirmed the association between OCT4 and TNM staging in other types of cancer, such as gastric cancer [9] and lung cancer [43]. Likewise, in gastric cancer, high levels of CSC biomarkers have been strongly associated with TNM staging, lymph node metastasis, and poor survival [44]. In contrast, Fujino et al. did not find a significant correlation between OCT4 expression and TNM staging; however, it is important to note that this study evaluated the expression of OCT4 mRNA, which was significantly correlated with poor metastasis-free survival [22].

There is evidence that points to the expression of OCT4 in the regulation of various signaling pathways associated with tumor formation and malignant transformation and increased recurrence, such as p38 mitogen-activated protein kinase (MAPK)/caspase-3, Wnt/β-catenin, AKT, and Janus Kinase (JAK)/signal transducer and activator of transcription (STAT)3 signal pathways [45,46,47]. Therefore, our results regarding the presence of OCT4 in RC at its various stages increase the possibility that CSCs are involved in resistance to conventional radiotherapy and chemotherapy treatments, increasing recurrences. It has previously been reported in patients with RC who underwent preoperative chemoradiotherapy that the CD133, OCT4, and SOX2 markers could be useful for predicting distant recurrence and poor prognosis, in addition to their possible association with tumor regrowth and metastases after chemoradiotherapy. [48]. Consequently, the data generated by our study could point towards the oncogenic role of OCT4 in RC, supported by the fact that the aberrant expression of this transcription factor and abnormal biological behavior of signaling pathways in stem cells during the development of RC may contribute to the promotion of tumorigenesis, its progression and aggressiveness, and the promotion of recurrences.

The major limitation of this study was the small sample size; however, we consider the obtained results valuable, taking into account the scarcity of recent data referring to clinicopathological and molecular aspects of RC in our population. We believe that a larger study population would allow us to validate our results; furthermore, it would be relevant to verify the data obtained through other molecular techniques, as well as to analyze the behavior of this marker over time, in order to analyze its clinical potential in the diagnosis, prognosis, and treatment follow-up in patients with RC.

## 5. Conclusions

We demonstrated expression of the stem cell marker OCT4 in tumor tissue of RC and the absence of modulation of this protein in adjacent non-tumor tissue; furthermore, we found that high expression of OCT4 was associated with undifferentiated histological grade, a greater degree of tumor invasion, lymph node involvement, and advanced or regional clinical stage, and higher TNM grades. Therefore, our results suggest that high expression of OCT4 is associated with a more aggressive phenotype of RC, with a greater likelihood of progression and metastasis. These findings shed light on the importance of focusing on this CSC marker and directing further studies aimed at investigating the mechanisms involved in its probable role in the initiation and progression of RC.

## Figures and Tables

**Table 1:** Sociodemographic, clinical, and pathological characteristics of the studied population.

Characteristics	*N* = 63	
	*n*	%
*Age*		
≤50 years	17	27
>50 years	46	73
*Sex*		
Female	38	60.3
Male	25	39.7
*Residential area*		
Urban	47	74.6
Rural	16	25.4
*Main sign/symptom*		
Rectal bleeding	33	52.4
Change in bowel habits	10	15.9
Weight loss	1	1.6
Anemia	2	3.2
Acute abdominal pain	10	15.9
Intestinal obstruction	6	9.5
Other	1	1.6
*Finding during colonoscopy*		
Ulcerative lesion	3	4.8
Multiple polyps	3	4.8
Single polyp	4	6.3
Exophytic lesion	36	57.1
Stenosing lesion	17	27
*Histological type*		
Adenocarcinoma	55	87.3
Mucinous adenocarcinoma	3	4.8
Neuroendocrine carcinoma	5	7.9
*Histological grade*		
Well-differentiated	24	38.1
Moderately differentiated	28	44.4
Poorly differentiated	11	17.5
*Clinical stage*		
Early/local	22	34.9
Advanced/regional	41	65.1
*TNM*		
0	1	1.6
I	8	12.7
IIA	7	11.1
IIB	3	4.8
IIC	4	6.3
IIIA	7	11.1
IIIB	14	22.2
IIIC	5	7.9
IVA	11	17.5
IVB	3	4.8
*Local invasion*		
TIs	4	6.3
T1	6	9.5
T2	21	33.3
T3	11	17.5
T4a	20	31.7
T4b	1	1.6
*Lymph node involvement*		
N1a	30	47.6
N1b	15	23.8
N1c	4	6.3
N2a	11	17.5
N2b	1	1.6
Unknown	2	3.2
*Metastasis*		
M0	49	77.8
M1a	11	17.5
M1b	3	4.8
*Vascular invasion*		
Si	15	23.8
No	29	46
Unknown	19	30.2

**Table 2:** Association between OCT4 expression and clinical and pathological variables of the studied samples (*n* = 22).

	Total Samples	OCT4 Expression		*p*-Value
		High	Low	
		*n* (%)	*n* (%)	
*Histological grade*				
Well-differentiated	8 (36.4)	4 (18.2)	4 (18.2)	0.039 *
Undifferentiated	14 (63.6)	13 (59.1)	1 (4.5)	
*Local invasion*				
TIs	2 (9.1)	0	2 (9.1)	0.004 **
T1	3 (13.6)	1 (4.5)	2 (9.1)	
T2	9 (40.9)	9 (40.9)	0	
T3	4 (18.2)	3 (13.6)	1 (4.5)	
T4a	3 (13.6)	3 (13.6)	0	
T4b	1 (4.5)	1 (4.5)	0	
*Lymph node involvement*				
N1a	12 (54.5)	7 (31.8)	5 (22.7)	0.044 *
N1b	2 (9.1)	2 (9.1)	0	
N1c	3 (13.6)	3 (13.6)	0	
N2a	4 (18.2)	4 (18.2)	0	
N2b	1 (4.5)	1 (4.5)	0	
*Metastasis*				
M0	20 (90.9)	15 (68.2)	5 (22.7)	0.458
M1a	1 (4.5)	1 (4.5)	0	
M1b	1 (4.5)	1 (4.5)	0	
*TNM*				
I	5 (22.7)	1 (4.5)	4 (18.2)	0.002 **
II	2 (9.1)	2 (9.1)	0	
III	13 (59.1)	12 (54.5)	1 (4.5)	
IVA	2 (9.1)	2 (9.1)	0	
*Clinical stage*				
Early/local	7 (31.8)	3 (13.6)	4 (18.2)	0.021 *
Late/regional	15 (68.2)	14 (63.6)	1 (4.5)	

* *p* < 0.05; ** *p* < 0.01.

## Data Availability

All data are contained within the manuscript. Raw data are available upon reasonable request from the corresponding author.

## Supplementary Materials

The following supporting information can be downloaded at: https://www.mdpi.com/article/10.3390/cancers15143740/s1. File S1: The original image of the Western Blots.

## References

[1] Ferlay J., Ervik M., Lam F., Colombet M., Mery L., Piñeros M., Znaor A., Soerjomataram I., Bray F. Global Cancer Observatory (GLOBOCAN): Cancer Today. https://gco.iarc.fr/today/data/factsheets/populations/900-world-fact-sheets.pdf.

[2] Schlechter B.L. (2022). Management of Rectal Cancer. Hematol Oncol. Clin. N. Am..

[3] Paschke S., Jafarov S., Staib L., Kreuser E.D., Maulbecker-Armstrong C., Roitman M., Holm T., Harris C.C., Link K.H., Kornmann M. (2018). Are Colon and Rectal Cancer Two Different Tumor Entities? A Proposal to Abandon the Term Colorectal Cancer. Int. J. Mol. Sci..

[4] Zhao X., Han P., Zhang L., Ma J., Dong F., Zang L., He Z., Zheng M. (2022). Prolonged neoadjuvant chemotherapy without radiation versus total neoadjuvant therapy for locally advanced rectal cancer: A propensity score matched study. Front. Oncol..

[5] Chen B., Sun H., Xu S., Mo Q. (2022). Long Non-coding RNA TPT1-AS1 Suppresses APC Transcription in a STAT1-Dependent Manner to Increase the Stemness of Colorectal Cancer Stem Cells. Mol. Biotechnol..

[6] Zhong L., Tan W., Yang Q., Zou Z., Zhou R., Huang Y., Qiu Z., Zheng K., Huang Z. (2022). PRRX1 promotes colorectal cancer stemness and chemoresistance via the JAK2/STAT3 axis by targeting IL-6. J. Gastrointest. Oncol..

[7] Yan Q., Fang X., Li C., Lan P., Guan X. (2022). Oncofetal proteins and cancer stem cells. Essays Biochem..

[8] Pandian J., Panneerpandian P., Sekar B.T., Selvarasu K., Ganesan K. (2022). OCT4-mediated transcription confers oncogenic advantage for a subset of gastric tumors with poor clinical outcome. Funct. Integr. Genom..

[9] Ibrahim D.A., Elsebai E.A., Fayed A., Abdelrahman A.E. (2022). Prognostic value of NOTCH1 and OCT4 in gastric carcinoma. Indian J. Pathol. Microbiol..

[10] Basati G., Mohammadpour H., Emami-Razavi A. (2020). Association of High Expression Levels of SOX2, NANOG, and OCT4 in Gastric Cancer Tumor Tissues with Progression and Poor Prognosis. J. Gastrointest. Cancer.

[11] Khoshchehreh R., Totonchi M., Ramirez J.C., Torres R., Baharvand H., Aicher A., Ebrahimi M., Heeschen C. (2019). Epigenetic reprogramming of primary pancreatic cancer cells counteracts their in vivo tumourigenicity. Oncogene.

[12] Jin X., Li Y., Guo Y., Jia Y., Qu H., Lu Y., Song P., Zhang X., Shao Y., Qi D. (2019). ERα is required for suppressing OCT4-induced proliferation of breast cancer cells via DNMT1/ISL1/ERK axis. Cell Prolif..

[13] Siddiqui Z., Srivastava A.N., Sankhwar S.N., Zaidi N., Fatima N., Singh S., Yusuf M. (2019). Oct-4: A prognostic biomarker of urinary bladder cancer in North India. Ther. Adv. Urol..

[14] Robinson M., Gilbert S.F., Waters J.A., Lujano-Olazaba O., Lara J., Alexander L.J., Green S.E., Burkeen G.A., Patrus O., Sarwar Z. (2021). Characterization of SOX2, OCT4 and NANOG in Ovarian Cancer Tumor-Initiating Cells. Cancers.

[15] Vaddi P.K., Stamnes M.A., Cao H., Chen S. (2019). Elimination of SOX2/OCT4-Associated Prostate Cancer Stem Cells Blocks Tumor Development and Enhances Therapeutic Response. Cancers.

[16] Lai S.C., Su Y.T., Chi C.C., Kuo Y.C., Lee K.F., Wu Y.C., Lan P.C., Yang M.H., Chang T.S., Huang Y.H. (2019). DNMT3b/OCT4 expression confers sorafenib resistance and poor prognosis of hepatocellular carcinoma through IL-6/STAT3 regulation. J. Exp. Clin. Cancer Res..

[17] Marques V., Ourô S., Afonso M.B., Rodrigues C.M.P. (2023). Modulation of rectal cancer stemness, patient outcome and therapy response by adipokines. J. Physiol. Biochem..

[18] Shao M., Bi T., Ding W., Yu C., Jiang C., Yang H., Sun X., Yang M. (2018). OCT4 Potentiates Radio-Resistance and Migration Activity of Rectal Cancer Cells by Improving Epithelial-Mesenchymal Transition in a ZEB1 Dependent Manner. Biomed. Res. Int..

[19] You L., Guo X., Huang Y. (2018). Correlation of Cancer Stem-Cell Markers OCT4, SOX2, and NANOG with Clinicopathological Features and Prognosis in Operative Patients with Rectal Cancer. Yonsei Med. J..

[20] Johari B., Rezaeejam H., Moradi M., Taghipour Z., Saltanatpour Z., Mortazavi Y., Nasehi L. (2020). Increasing the colon cancer cells sensitivity toward radiation therapy via application of Oct4-Sox2 complex decoy oligodeoxynucleotides. Mol. Biol. Rep..

[21] Roudi R., Barodabi M., Madjd Z., Roviello G., Corona S.P., Panahi M. (2020). Expression patterns and clinical significance of the potential cancer stem cell markers OCT4 and NANOG in colorectal cancer patients. Mol. Cell Oncol..

[22] Fujino S., Miyoshi N. (2019). Oct4 Gene Expression in Primary Colorectal Cancer Promotes Liver Metastasis. Stem Cells Int..

[23] Edge S.B., Byrd D.R., Compton C.C., Fritz A.G., Greene F.L., Trotti A., American Joint Committee on Cancer (2010). Colon and Rectum. AJCC Cancer Staging Manual.

[24] Kruger N.J., Walker J.M. (2009). The Bradford Method for Protein Quantitation. The Protein Protocols Handbook.

[25] Sambrook J., Fritsch E., Maniatis T. (1989). Molecular Cloning: A Laboratory Manual, 2nd. ed..

[26] Moreno-Londoño A.P., Castañeda-Patlán M.C., Sarabia-Sánchez M.A., Macías-Silva M., Robles-Flores M. (2023). Canonical Wnt Pathway Is Involved in Chemoresistance and Cell Cycle Arrest Induction in Colon Cancer Cell Line Spheroids. Int. J. Mol. Sci..

[27] Vasefifar P., Motafakkerazad R., Maleki L.A., Najafi S., Ghrobaninezhad F., Najafzadeh B., Alemohammad H., Amini M., Baghbanzadeh A., Baradaran B. (2022). Nanog, as a key cancer stem cell marker in tumor progression. Gene.

[28] Boman B.M., Viswanathan V., Facey C.O.B., Fields J.Z., Stave J.W. (2023). The v8-10 variant isoform of CD44 is selectively expressed in the normal human colonic stem cell niche and frequently is overexpressed in colon carcinomas during tumor development. Cancer Biol. Ther..

[29] Frau C., Jamard C., Delpouve G., Guardia G.D.A., Machon C., Pilati C., Nevé C.L., Laurent-Puig P., Guitton J., Galante P.A.F. (2021). Deciphering the Role of Intestinal Crypt Cell Populations in Resistance to Chemotherapy. Cancer Res..

[30] Pádua D., Figueira P., Ribeiro I., Almeida R., Mesquita P. (2020). The Relevance of Transcription Factors in Gastric and Colorectal Cancer Stem Cells Identification and Eradication. Front Cell Dev. Biol..

[31] Lumsdaine C.T., Liu-Smith F., Li X., Zell J.A., Lu Y. (2020). Increased incidence of early onset colorectal adenocarcinoma is accompanied by an increased incidence of rectal neuroendocrine tumors. Am. J. Cancer Res..

[32] Vergara E.E., Núñez G.A., Hoyos J.C., Lozada-Martínez I.D., Suarez A., Narvaez-Rojas A.R. (2023). Surgical outcomes and factors associated with postoperative complications of colorectal cancer in a Colombian Caribbean population: Results from a regional referral hospital. Cancer Rep..

[33] Vaccaro C.A., López-Kostner F., Adriana D.V., Palmero E.I., Rossi B.M., Antelo M., Solano A., Carraro D.M., Forones N.M., Bohorquez M. (2019). From colorectal cancer pattern to the characterization of individuals at risk: Picture for genetic research in Latin America. Int. J. Cancer.

[34] Mauri G., Sartore-Bianchi A., Russo A.G., Marsoni S., Bardelli A., Siena S. (2019). Early-onset colorectal cancer in young individuals. Mol. Oncol..

[35] Siegel R.L., Wagle N.S., Cercek A., Smith R.A., Jemal A. (2023). Colorectal cancer statistics, 2023. CA Cancer J. Clin..

[36] Musetti C., Garau M., Alonso R., Piñeros M., Soerjomataram I., Barrios E. (2021). Colorectal Cancer in Young and Older Adults in Uruguay: Changes in Recent Incidence and Mortality Trends. Int. J. Environ. Res. Public Health.

[37] Vargas-Moranth R., Navarro-Lechuga E. (2018). Cancer incidence and mortality in Barranquilla, Colombia. 2008–2012. Colomb. Med..

[38] Shaheen M.A., Hegazy N.A., Nada O.H., Radwan N.A., Talaat S.M. (2014). Immunohistochemical expression of stem cell markers CD133 and Oct4 in colorectal adenocarcinoma. Egypt J. Pathol..

[39] Pece S., Tosoni D., Confalonieri S., Mazzarol G., Vecchi M., Ronzoni S., Bernard L., Viale G., Pelicci P.G., di Fiore P.P. (2010). Biological and molecular heterogeneity of breast cancers correlates with their cancer stem cell content. Cell.

[40] Brown R.E., Ali Y.D., Cai Z. (2020). Morphoproteomics Identifies CXCR4 in Undifferentiated Colorectal Cancer: A Case Study with Therapeutic Implications. Ann. Clin. Lab. Sci..

[41] Gao Z.-Y., Liu X.-B., Yang F.-M., Liu L., Zhao J.-Z., Gao B., Li S.-B. (2019). Octamer binding transcription factor-4 expression is associated with cervical cancer malignancy and histological differentiation: A systematic review and meta-analysis. Biosci. Rep..

[42] Mohamed S.Y., Kaf R.M., Ahmed M.M., Elwan A., Ashour H.R., Ibrahim A. (2019). The Prognostic Value of Cancer Stem Cell Markers (Notch1, ALDH1, and CD44) in Primary Colorectal Carcinoma. J. Gastrointest. Cancer.

[43] Li H., Wang L., Shi S., Xu Y., Dai X., Li H., Wang J., Zhang Q., Wang Y., Sun S. (2019). The Prognostic and Clinicopathologic Characteristics of OCT4 and Lung Cancer: A Meta-Analysis. Curr. Mol. Med..

[44] Razmi M., Ghods R., Vafaei S., Sahlolbei M., Saeednejad-Zanjani L., Madjd Z. (2021). Clinical and prognostic significances of cancer stem cell markers in gastric cancer patients: A systematic review and meta-analysis. Cancer Cell Int..

[45] Zhang Q., Han Z., Zhu Y., Chen J., Li W. (2020). The role and specific mechanism of OCT4 in cancer stem cells: A review. Int. J. Stem Cells.

[46] Xie W., Yu J., Yin Y., Zhang X., Zheng X., Wang X. (2022). OCT4 induces EMT and promotes ovarian cancer progression by regulating the PI3K/AKT/mTOR pathway. Front Oncol..

[47] Guo K., Duan J., Lu J., Xiao L., Han L., Zeng S., Tang X., Li W., Huang L., Zhang Y. (2022). Tumor necrosis factor-α-inducing protein of Helicobacter pylori promotes epithelial-mesenchymal transition and cancer stem-like cells properties via activation of Wnt/β-catenin signaling pathway in gastric cancer cells. Pathog. Dis..

[48] Saigusa S., Tanaka K., Toiyama Y., Yokoe T., Okugawa Y., Ioue Y., Miki C., Kusunoki M. (2009). Correlation of CD133, OCT4, and SOX2 in rectal cancer and their association with distant recurrence after chemoradiotherapy. Ann. Surg. Oncol..

