# Supplementary material for: High OCT4 Expression Might Be Associated with an Aggressive Phenotype in Rectal Cancer
Source: Cancers (Basel). 2023 Jul 23;15(14):3740. doi: 10.3390/cancers15143740 (PMC10378144; doi:10.3390/cancers15143740)
Supplement: Supplementary file 1 [file cancers-15-03740-s001.zip › cancers-2483103-supplementary.pdf]

Supplementary File S1. The original image of the Western Blots.

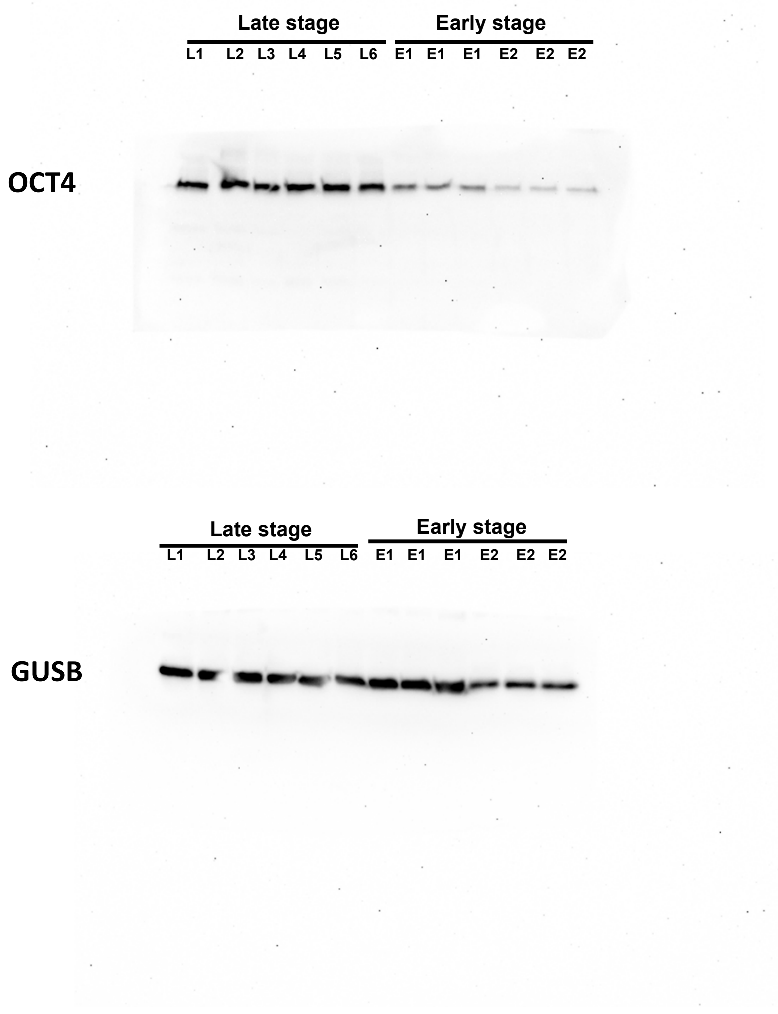

Samples L1 - L6 correspond to late-stage rectal tumor tissue. Samples E1 and E2 correspond to early-stage rectal tumor tissue.

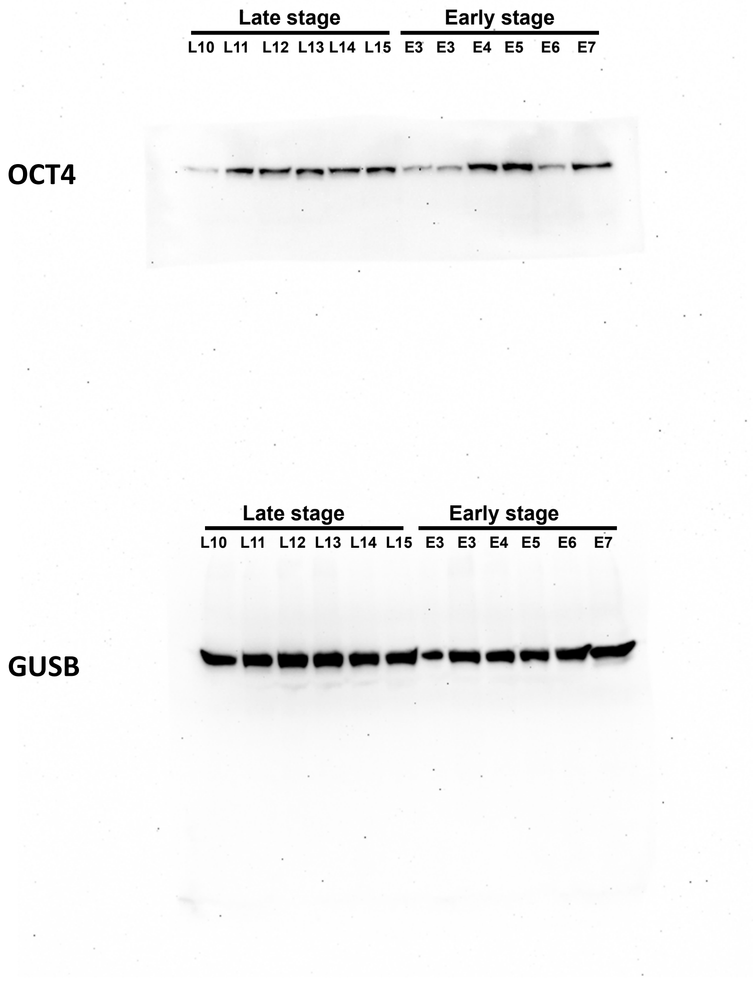

Samples L10 - L15 correspond to late-stage rectal tumor tissue. Samples E3 - E7 correspond to early-stage rectal tumor tissue.

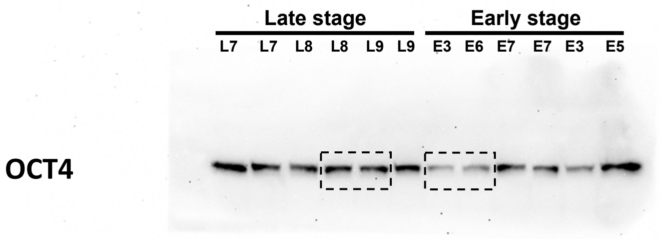

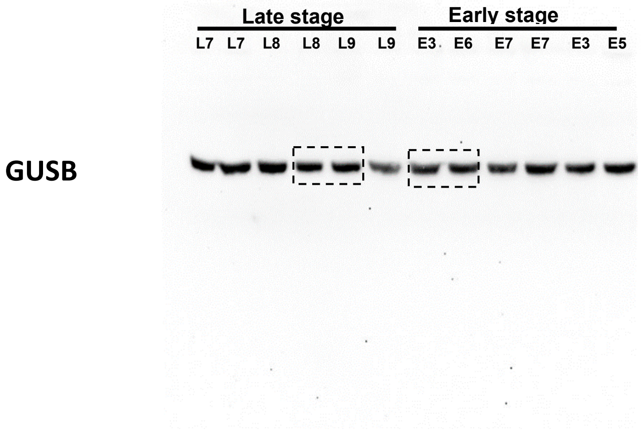

Samples L7, L8 and L9 correspond to late-stage rectal tumor tissue. Samples E3, E6, E7 and E5 correspond to early-stage rectal tumor tissue. The dotted rectangles delineate the areas shown in Figure 1c.

OCT4

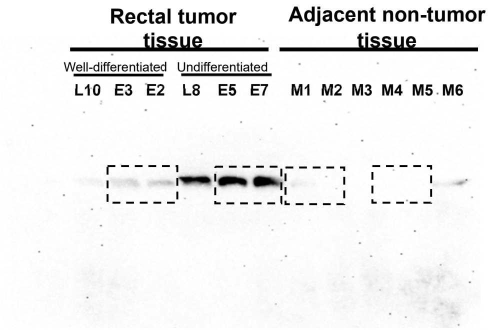

GUSB

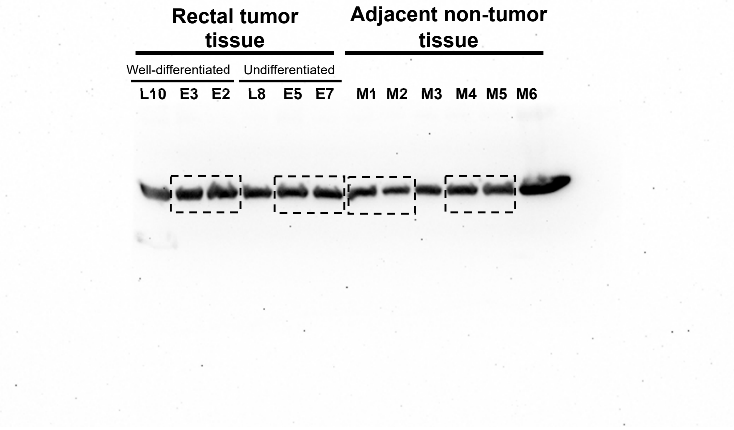

Samples L10, E3 and E2 correspond to well-differentiated rectal tumor tissue; samples L8, E5 and E7 correspond to undifferentiated rectal tumor tissue. Samples M1 – M6 correspond to adjacent non-tumor tissue. The dotted rectangles delineate the areas shown in Figure 1a and 1c.
